# Organ Contouring for Lung Cancer Patients with a Seed Generation Scheme and Random Walks

**DOI:** 10.3390/s20174823

**Published:** 2020-08-26

**Authors:** Da-Chuan Cheng, Jen-Hong Chi, Shih-Neng Yang, Shing-Hong Liu

**Affiliations:** 1 Department of Biomedical Imaging and Radiological Science, China Medical University, Taichung City 40402, Taiwan; dccheng@mail.cmu.edu.tw; 2 Department of Diagnostic Radiology, Singapore General Hospital, Singapore 169608, Singapore; chi811210@gmail.com; 3 Department of Radiation Oncology, China Medical University Hospital, Taichung City 40447, Taiwan; 4 Department of Computer Science and Information Engineering Chaoyang University of Technology, Taichung City 41349, Taiwan

**Keywords:** radiotherapy planning, organ contouring, interactive segmentation

## Abstract

In this study, we proposed a semi-automated and interactive scheme for organ contouring in radiotherapy planning for patients with non-small cell lung cancers. Several organs were contoured, including the lungs, airway, heart, spinal cord, body, and gross tumor volume (GTV). We proposed some schemes to automatically generate and vanish the seeds of the random walks (RW) algorithm. We considered 25 lung cancer patients, whose computed tomography (CT) images were obtained from the China Medical University Hospital (CMUH) in Taichung, Taiwan. The manual contours made by clinical oncologists were taken as the gold standard for comparison to evaluate the performance of our proposed method. The Dice coefficient between two contours of the same organ was computed to evaluate the similarity. The average Dice coefficients for the lungs, airway, heart, spinal cord, and body and GTV segmentation were 0.92, 0.84, 0.83, 0.73, 0.85 and 0.66, respectively. The computation time was between 2 to 4 min for a whole CT sequence segmentation. The results showed that our method has the potential to assist oncologists in the process of radiotherapy treatment in the CMUH, and hopefully in other hospitals as well, by saving a tremendous amount of time in contouring.

## 1. Introduction

Radiotherapy is often used as the main treatment for lung cancer. Some patients with lung cancer might choose to receive radiotherapy [1]. Others might choose radiotherapy after surgery, or after chemotherapy. Radiotherapy uses high-energy radiation to kill tumor cells. However, radiation can destroy not only tumor cells, but also normal tissues during treatment. Therefore, a Radiotherapy Treatment Plan (RTTP), which is used to distinguish tumors from normal organs, needs to be designed before radiation is carried out. An RTTP will simulate the dose distribution of the organs which may take radiation during radiotherapy [2]. Needless to say, this therapy planning is crucial. Oncologists in the Chinese Medical University Hospital (CMUH) are required to perform RTTPs regularly. The contouring work in an RTTP is traditionally performed manually by experienced radiation oncologists. This process usually takes more than half an hour to two hours for a case, depending on the performance of the commercial product (in our study, Eclipse) and the experience of the oncologists. Until now, there has been no fully automated contouring commercial product for all organs and tumors. We believe that some contouring automation would offer considerable assistance in developing RTTPs.

Target delineation is basically an organ contouring task. In the field of medical image segmentation, either manually or automatically, it is a non-trivial task to delineate organs which are similar to their neighboring ones. Experts can perform the task manually because they have professional knowledge. For instance, the spleen and liver are near to each other and have similar X-ray attenuation coefficients. A tumor is a soft and abnormal tissue. Usually, lung tumors are easy to visually observe on computed tomography (CT) if their sizes are large enough. Experienced oncologists can delineate a lung tumor manually based on CT or positron emission computed tomography (PET) evidence, and some other clinical evidence. Image segmentation methods may be region-based [3], edge-based [4,5], or hybrid [6]. A semi-automated system allows the user to incorporate pre-defined constraints [7], or provide the interaction with the segmentation result. This interaction makes the image segmentation more convenient against the variations between patients.

The goal of this study was to design a semi-automated software system for contouring four organs, the body, and tumors based on CT images in RTTPs, with less human intervention. The random walks (RW) algorithm was used to perform image segmentation [8]. Some schemes were proposed to generate good seeds and to remove bad seeds. These schemes leveraged anatomical knowledge, described in Section 2.2, Section 2.3 and Section 2.4. First, we manually set up “seeds” on an image, which had the category labels and position information. Then, the RW algorithm calculated the class probabilities of the rest of the “unseeded” points on the same image. With our schemes, the good seeds survived and the bad seeds vanished in the following process. The seeds were obtained first manually and then were generated automatically by our schemes slice-by-slice. With less human intervention, the contours of organs could be delineated by our system. Except for tumor contours, the contours of critical organs could be generated within an acceptable error rate.

This paper is organized as follows. The materials and methods are introduced in Section 2. The results of organ contouring, qualitative comparisons, and system validity are shown in Section 3. In Section 4 we include the discussion. Finally, we outline the conclusions in Section 5.

## 2. Materials and Methods

### 2.1. Materials

The hardware was a personal computer with an Intel Core i5-3230M CPU @ 2.6 GHz and 16 GB memory. A graphical user interface (GUI) was created using Matlab (MathWorks, Natick, MA, USA) [9] for easy operation. We considered 25 patients with non-small cell lung cancers, whose CT images were obtained from the China Medical University Hospital. The data were collected from two different CT scanners in two different years in order to test the robustness of the developed software. The first dataset had 15 patients and was collected in 2011 by the GE^®^ CT machine (HISPEED NX/I, Aventura, FL, USA). The slice number of each image sequence was from 61 to 136. The slice thickness was 2.5 mm, and the pixel size was 1.04 mm^2^. The second dataset had 10 patients and was collected in 2017 by a SIEMENS^®^ CT machine (SOMATOM Definition AS, Berlin, Germany). The slice thickness was 2 mm and the pixel size was 0.95 mm^2^. The image spatial resolution was 512 × 512 pixels. The age range of patients was 50 to 86 years old. This retrospective research was approved by the Research Ethics Committee of China Medical University and Hospital, Taichung, Taiwan, with the protocol number CMUH106-REC3-015.

Before segmentation, all grey-levels were adjusted to the window of soft tissue, [−140, 260] HU, by a linear transformation. Values lower or higher than this range were set to 0 or 255, separately. This image enhancement process was used not only for easy visualization, but also for easy organ contouring.

### 2.2. Initial Settings

**Figure 1:**
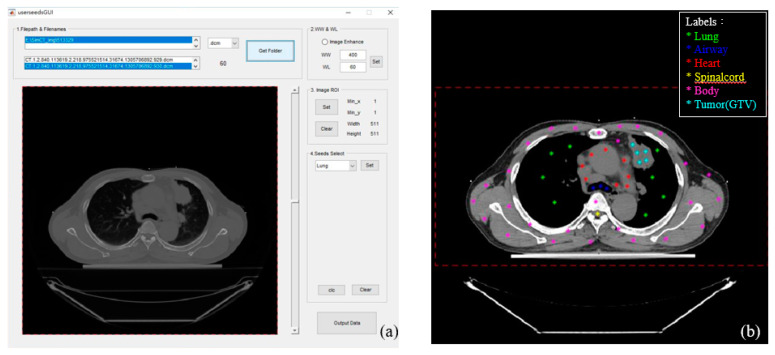
(**a**) The easy-to-operate GUI for users. (**b**) Initial seeds given by the user. A different color means a different category: lungs (green), airway (blue), heart (red), spinal cord (yellow), body (pink), the GTV (green-blue). The heart category contains the heart and its neighboring blood vessels (part of mediastinum having a similar HU to the heart).

The GUI was designed to perform five tasks: (1) selecting the image data directory; (2) setting the appropriate rectangle region of interest (ROI), as shown in Figure 1b; (3) inputting some initial seeds on six categories; (4) defining the slice numbers where the heart appears and disappears; and (5) defining the initial slice locating the junction of the trachea and the bronchus.

The number of seeds on each category was from 1 to 20 in the experiments, depending on the size of the organ categories. After being set, the software system would segment the organs and outline the contours efficiently. Notably, the seeds were a manual input in the initial setting (Section 2.2). However, in the remaining parts (Section 2.3, Section 2.4, Section 2.5 and Section 2.6), the seeds were automatically generated. The GUI allowed users to give seeds in one or more slices in the beginning. However, in order to maintain the same conditions to evaluate the performance of our software system, we set initial seeds only on one slice in our experiments. We did not correct the contour delineations through all experiments, although our software system was interactive.

### 2.3. Random Walks Algorithm

The RW was proposed by Leo Grady in 2006 [8]. The user gives several point coordinates and defines their labels (we called them seeds). After solving the linear equation system, the probability of each pixel (position) is calculated. The pixels are assigned to the label which has the highest probability at the pixel to perform region classification, which is the result of image segmentation. The RW had been widely used with many medical images made by different modalities, such as CT [10], magnetic resonance imaging (MRI) [11], sonography [12], and PET [13]. The advantages of using RW in processing medical images are twofold: (1) it is semi-automatic and interactive, and (2) it has a good performance with weak edges, which often exist in medical images [10]. In this study, we applied a semi-automatic procedure instead of a full-automatic procedure, as physicians prefer an algorithm which is interactive with human intervention so that they can fine-tune the initial conditions to improve the results, and because weak edges usually feature in medical images. For example, the X-ray based modality has a similar Hounsfield unit (HU) when two different organs or targets have a similar density. When these two different targets are close to each other, it is hard to separate them. Since RW needs initial seeds, the seeds define their correct labels so that the weak edges can be contoured accurately. The method for retaining/producing good seeds and removing bad seeds is described in Section 2.4 and Section 2.5.

The RW algorithm is based on graph theory, and segmentation is performed via minimizing the Dirichlet integral:

(1) $$D\left[u\right]=\frac{1}{2}\int_{\Omega }{\left|\nabla u\right|}^{2}d\Omega$$

where *u* is a 2D function representing an image, and Ω is a region representing the same label area. The segmentation problem is performed via minimizing the integral of the same labeling area, which is assumed to be a homogeneous area. The boundaries are excluded from the integral and are assumed to be gradients (maximal values) of the image gray-level. In order to minimize this, the integral must satisfy the Euler-Lagrange equation, which solves the Laplace equation:

(2) $$\nabla^{2}u=0.$$

Since the image function *u* is discrete, the combinatorial matrix is used to replace the continuous function. Thereafter, the Dirichlet integral is transformed to solve a combinatorial formulation of the Dirichlet integral:

(3) $$D[x]=\frac{1}{2}{\left(Ax\right)}^{T}C(Ax)=\frac{1}{2}x^{T}Lx=\frac{1}{2}\sum_{e_{i,j}\in E}\omega_{ij}{\left(x_{i}-x_{j}\right)}^{2}$$

where *L* is the combinatorial Laplacian matrix; *A* is the edge-node incidence matrix; and *C* is the constitutive matrix. *x* represents a harmonic function. It can calculate the class probability of the rest of the points with some given limited class-labeled seeds, via the minimization process in Equation (1). Based on the class probability, one can determine the pixel belonging to the class with the largest probability. In RW, there is only one free parameter, *β*, which affects the Gaussian weighting factor *ω_ij_,* defined as follows:

(4) $$\omega_{ij}=\exp(-\beta {\left(g_{i}-g_{j}\right)}^{2})$$

where *g_i_* and *g_j_* are the pixel gray levels at pixel *i* and *j*. Readers can refer to work of Grady [8] for details. In this study, we set *β* = 70. We undertook some additional experiments, and address the reason for this setting in Section 3.

### 2.4. Boundary Erosion

The algorithm started from the slice where the initial seeds were given. Contours were delineated, including the lungs, airway, heart (including neighboring blood vessels), spinal cord, body, and the GTV (tumor). The slice number where the junction of the trachea and the bronchus was located was used to guide the algorithm for the separation of the bronchus on the inferior slice.

Usually, human organ contours do not change suddenly when the CT slice thickness is small (2 mm or 2.5 mm). We used the segmentation results of the previous slice as guidance. However, we eroded the previous segmentation result to prevent potential bad seeds. A few points on the eroded edge of the segmentation on the previous slice were automatically sampled to be the seeds of the current slice. The element size of the erosion structure was important [14]. Since the targets were quite different in size, they needed different radii in the erosion. The large targets (areas larger than 1000 pixels, such as the lungs, heart, body, etc.) were eroded in a disk shape, with a radius = 12 pixels. The rest of the small targets, such as the spinal cord and airway, were eroded with radius = 1 pixel. In addition, some targets were small at the top or bottom position, and some targets were thin. In these cases, it was possible that seeds would be removed by the erosion process, and no seeds would be retained. To solve this seeds-vanishing problem, we proposed another strategy; this is outlined in the next section.

### 2.5. Use of the Skeleton Technique to Produce Seeds

In most cases, the seeds could be retained in the same target by the erosion process. However, if the segmented target was thin (such as the chest muscle region), the whole region would possibly be removed and the seeds would vanish. To solve this problem, we proposed using the skeleton technique or thinning technique [14,15] to produce some seeds. The thinning process was performed on all segmented regions in the previous slice. After the thinning process, a pruning algorithm was performed to prune branch ends to produce a better skeleton [15]. Afterward, a few points (defined by a given ratio of the whole skeleton point number) on the skeleton were randomly sampled to produce new seeds. In summary, there were two sources for generating seeds: (1) those sampled from an eroded boundary, and (2) those sampled from the skeleton. However, it was possible that a few seeds were located in a different organ on the next slice. These seeds were called bad seeds. For example, the spine-seeds were located outside the spine area in the next slice. This rare case might happen when the target vanishes or changes its position. In order to prevent the appearances of these cases, all seeds with the same label were checked to see whether the HU of their position matched the correct label. Our strategy used the organ information to check the seeds. A knowledge pool of anatomic HU information was built beforehand. The HU table was also transformed into a gray-level table using the same procedure as described in the last paragraph in Section 2.1. For example, the lung and airway seeds had small gray levels ranging from 0 to 10, because they contained air. On the other hand, the body and tumor seeds must exclude those seeds which have gray levels less than 10. The spinal cord must exclude seeds with a 255 gray level, which may belong to bone tissue. With the process of the gray-level table of organs, some erroneous (bad) seeds were removed. The HU table is summarized in Table 1. Based on these strategies, the robustness of the system could be greatly improved.

### 2.6. Modification of the Spinal Cord Boundary

The boundary of the spinal cord on the thoracic vertebra is quasi round. In order to alleviate the detection problem on the spinal cord, we applied Hough transform circle fitting [14]. The advantage of this technique is that it can be operated under the interference of noise and outliers by using a voting process. In clinics, physicians can use a circle to fit the spinal cord, at the thoracic vertebrae, in contouring. The center of the spinal cord might shift slice-by-slice; therefore, it is necessary to fit the spinal cord slice-by-slice with an updated small ROI. The Sobel operator was applied in the ROI to get the edge points for the following Hough transform circle fitting [14].

### 2.7. System Accuracy

The gold standard for the delineation was given by physicians on Eclipse for comparison. To compare the semi-automated delineation and manual delineation, we used Dice coefficient (*DIC*) [16,17], defined as follows:

(5) $$DIC=\frac{2TP}{FP+2TP+FN}$$

where *TP*, *FP*, and *FN* denoted true positive, false positive, and false negative. The Dice coefficient measures the similarity of two sets. A *DIC* value approaching 1 meant a good degree of similarity.

## 3. Results

### 3.1. Initial Settings

Figure 1 shows the results of Section 2.2. Figure 1a shows the GUI, and Figure 1b shows the initial seeds given manually. The dots in different colors denote seeds in different categories. There are six categories: (1) lungs (green), (2) airway (blue), (3) heart (red), (4) spinal cord (yellow), (5) body (pink), and (6) the GTV (green-blue). The heart category contains the heart and its neighboring blood vessels. Notably, the initial seed number for the spinal cord needed only one seed. The dashed rectangle encompassing the body is the ROI, which is used to limit the computation area in order to speed up the whole process. The ROI is first suggested by the system, and the user can interact with it to change the size of the suggested ROI.

**Figure 2:**
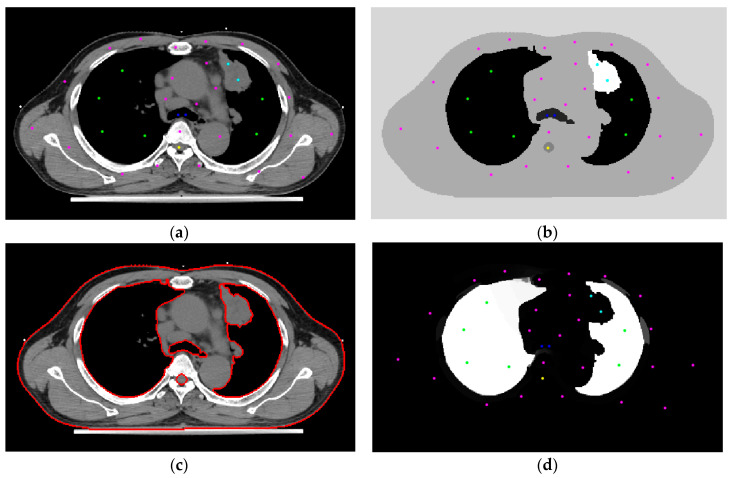
(**a**) The raw image with some initial seeds. (**b**) The RW segmentation result. (**c**) The automated delineations superimposed on the raw image. (**d**) The segmented lung area with seeds superimposed on the result.

Figure 2 denotes the details of processing one image segmentation using RW. Figure 2a is the raw image with some initial seeds superimposed onto it. The system starts from the image and does the image segmentation automatically. Figure 2b shows the result of the RW, with the initial seeds superimposed on the image. The white part is the tumor. Figure 2c shows contours of different categories, in which the spinal cord is fitted by a circle model using the Hough transform. Figure 2d is the segmentation result, and the white part is the lung.

### 3.2. Automated Segmentation

**Figure 3:**
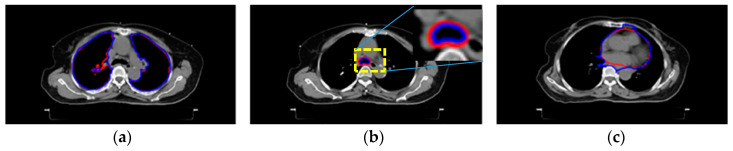

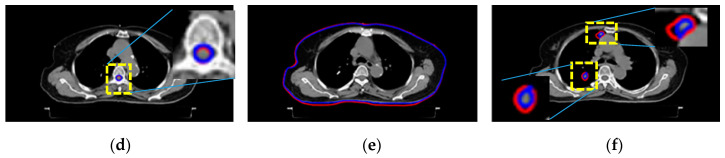
Qualitative comparison with manual segmentation results. Different categories are shown below: (**a**) lungs, (**b**) airway, (**c**) heart, (**d**) spinal cord, (**e**) body, and (**f**) tumor. Red and blue delineations denote manual and automated results, respectively.

Figure 3 shows the results of Section 2.4 and Section 2.5. Figure 3a–f show different targets’ segmentation results. These are the lungs, airway, heart, spinal cord, body, and tumors, respectively. The blue delineations are automated results, whereas the red lines were made by the radiologist. For comparison, these two results are superimposed on the raw image. All segmentation was done using automatic seed selection, as described in Section 2.4 and Section 2.5.

**Figure 4:**
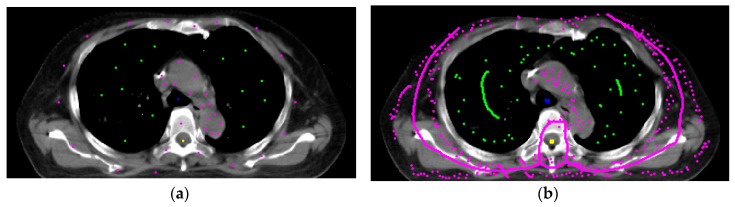
The seed generation and vanishing results. (**a**) The initial settings for seeds of different categories. (**b**) The automatic seed generation and vanishing on the next slice (superior direction).

**Figure 5:**
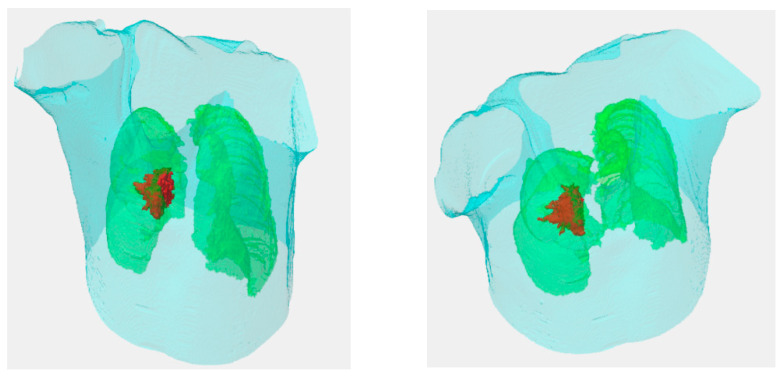
The 3D visualizations of the body shape, lungs, and GTV. The cyan denotes the body shape, green denotes the lungs, and the red denotes the GTV.

Figure 4a,b show the seeds’ automatic generation and vanishing results. The 2D RW processes each image slice-by-slice. Figure 4a is the initial settings of the seeds, and Figure 4b is the automatic seed generation on its next slice. From Figure 4b we see some seeds forming a ‘curve’, which is generated from the skeleton scheme described in Section 2.5. After segmentation, contours of all categories are obtained. Figure 5 show two views of the 3D visualizations of one patient. The cyan color is the body shape, the green color denotes the lungs, and the red color shows the GTV.

### 3.3. Free Parameter β

**Figure 6:**
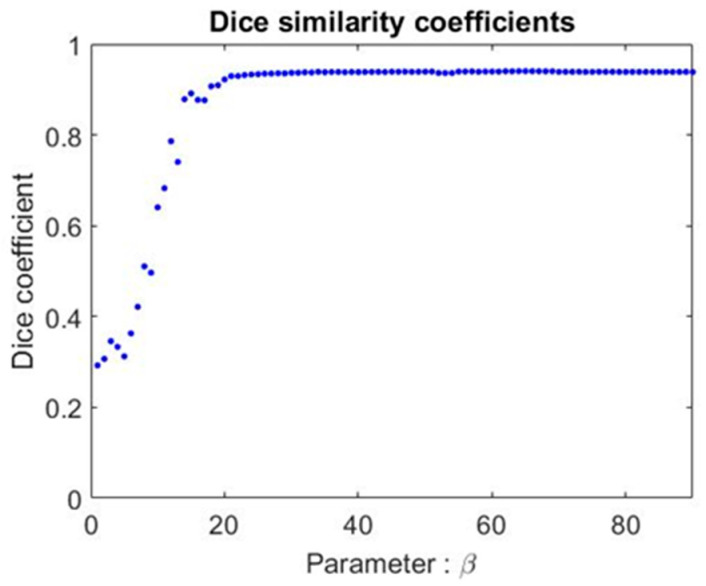
The Dice coefficient using different *β* values under the contouring of the lung. The two sets for comparison of the similarities are our automated contouring and the expert’s manual delineations.

To understand the influence of the free parameter *β*, we chose the lung as the target for comparison, and used the Dice coefficient to see the difference between our automated segmentation result and the expert’s manual drawing. The experiment was undertaken on one patient’s image sequence. The Dice coefficient is shown in Figure 6. From the results, we found that there was no change in the Dice coefficient when *β* was larger than 30. Since the original paper [8] suggested using *β* = 90, we compromised and set *β* = 70. The free parameter was not critical and not sensitive to results when its value was larger than 30 in this study.

### 3.4. System Validity

To assess the system validity, we computed the Dice coefficient between the two contours (physician’s manual and automated delineations) of the RTTP to evaluate their similarity. The average Dice coefficient results of the lungs, airway, heart, spinal cord, body, and GTV are listed in Table 2 and Table 3. The first dataset was collected from 15 patients using a GE^®^ CT machine (HISPEED NX/I, Aventura, FL, USA). The average Dice coefficients of the six targets were: 0.94, 0.86, 0.88, 0.74, 0.85, and 0.57. The computation time is listed in the last column and was dependent on the number of CT slices. The computation time was about 83 to 231 s, and the average time was 141 ± 48 s. Notably, the computation time does not include the initial manual settings. The second dataset was collected from 10 patients using a SIEMENS^®^ CT machine. The average Dice coefficients of the six targets were 0.90, 0.81, 0.78, 0.71, 0.84, and 0.78. The computation time was similar to the first experiment and is not shown in Table 2.

### 3.5. System Comparison

The commercial product used in CMUH is Eclipse (Version 13.6, Varian Medical Systems, Inc., Palo Alto, CA, USA). It is able to delineate only the lungs, spinal cord, and body automatically. In order to compare its performance, we used the Dice index, false-positive rate (FPR), and false-negative rate (FNR), as listed in Table 4. Two comparisons were performed; one between the gold standard (physician’s manual drawings) and the commercial product Eclipse (row 1 and 3), and another one between the gold standard (physician’s manual drawings) and our system (row 2 and 4). Our system can produce contours and transfer them to the format for Eclipse. Thus, follow-up planning can be readily performed. From Table 4, we can see that there are limited differences between our system and Eclipse. We admit that our results in the lung and spinal cord delineations are not as good as Eclipse. However, our system can contour six categories simultaneously. Except for the GTV, our system does not need manual correction; its performance is shown in Table 2 and Table 3.

### 3.6. Radiotherapy Plan

**Figure 7:**
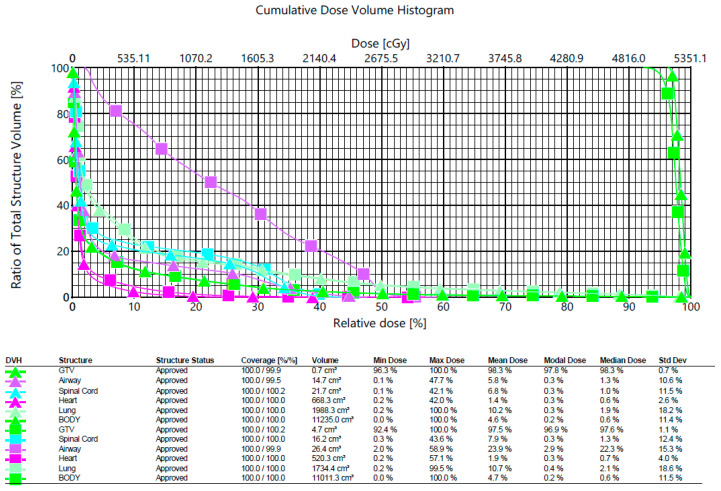
The radiation dose volume histogram. This result was computed by the commercial product (Eclipse) with the contour segmentation ready. The triangle marks denote the physician’s manual segmentation result, whereas the square marks denote the segmentation result by our system.

The contour delineations of the six categories can be input to Eclipse to compute the radiation dose histogram, as shown in Figure 7. We used one case as an example to see the difference between our segmentation results (the square marks) and the gold standard (physician’s manual segmentation, the triangle marks). The color denotations are listed below: green, purple, light blue, pink, light green, and Kelly green; corresponding to GTV, airway, spinal cord, heart, lung, and body, respectively. The abscissa denotes the cumulative dose, and the ordinate denotes the total structure volume. From the comparison, we can see that, with the exception of the airway, most categories are very similar. Our system’s contouring on the airway (purple square marks) absorbed as much of the radiation dose as the expert’s manual delineations (purple triangle). Both GTVs absorbed large radiation doses with these methods.

## 4. Discussion

In the CMUH, all normal organs and GTV are defined by physicians on CT images using Eclipse. Eclipse can complete lung, spinal cord, and body image segmentation fully automatically. However, the other normal organs (heart, airway) contours and the GTV have to be delineated manually by physicians. This is a time-consuming process because there are many image slices to delineate (usually more than 100 images per patient). Based on a physician’s experience, as we have mentioned before, the manual process might take from 30 min to two hours. This time range was obtained from a consensus of the oncologists in the CMUH. Counting the time spent on an RTTP by an oncologist is particularly difficult, for the following reasons:

(1) Every patient has different disease conditions. Their tumors, bodies, organs, and so forth are variable. An oncologist manages many patients and has to finish many RTTPs per day. Some patients are easy, and some are very challenging to handle.
(2) In some critical cases, one oncologist might need help from other more experienced oncologists. In such a situation, the time needed is increased.

In order to shorten the manual delineation process, we developed this software system. The initial input process requires about one to three minutes, and the automatic organ contouring process takes a further two to four minutes. The differences in the time spent depend on the number of images to be processed. The total process time is within ten minutes. After segmentation, all the contour coordinates can be input into Eclipse for further radiation dose computation to finish the RTTP.

In computer science, there are benchmarks for different algorithms to compare. The CIFAR-10 and CIFAR-100 [18] and Coco datasets [19] are some examples. These datasets provide more than 60,000 images, and represent the gold standard for comparison. A lot of deep learning methods [20,21] have been compared based on many kinds of benchmarks [18,19,22,23]; for example, handwritten digits [24] and lung nodule detection [25]. However, these cases were not applied in this study. In the case of lung cancer radiotherapy plans, there is no such benchmark. It is difficult to make comparisons between different algorithms. The most frequent way to undertake a comparison of performance is by using the dataset in-house and the gold standards, made manually by physicians in-house [26]. A similar situation was described in the author’s research [27]. These authors had a dataset in-house (cancer cell nucleus images), and they compared these images with manual counting. Another difficulty is that fully automatic organ contouring is not always reliable in clinics. Oncologists need to correct the automatic contouring interactively when the results are not acceptable. Generally, the oncologist can accept the assisted performance of an interactive approach. This approach is a good solution in clinical RTTPs.

In order to assess the system performance, we used the Dice coefficient to compare the segmentation results of our system with physician’s manual drawings (gold standard). The average Dice coefficients for the lungs, airway, heart, spinal cord, body and GTV were 0.92, 0.84, 0.83, 0.73, 0.85 and 0.66, respectively. Except for the spinal cord and GTV, most normal organs are well-segmented (Dice coefficients larger than 0.8). In our system, the contour of the spinal cord was slightly overestimated (area was slightly larger than the physician’s definition).

The Hausdorff distance (HD) [28] is a metric to measure the distance between two sets. More formally, the Hausdorff distance is defined as:

(6) $$h\left(A,B\right)=\underset{a\in A}{\max}\left\{\underset{b\in B}{\min}d\left(a,b\right)\right\}$$

As mentioned in Ghaffari’s paper [29], “HD suffers from the so-called panhandle problem, which occurs when one object exhibiting a sudden local shape deviation causes an unrealistically large HD value.” In order to prevent the panhandle problem, we prefer to use the Dice coefficient. However, the Dice similarity has another problem, as mentioned by Ghaffari et al. [29]: “this assessment does not allow direct comparison over different length-scales”. In other words, the same Dice coefficient value for a large area does not have the same accuracy as in a small area.

Clinical radiation oncologists or physicians use not only CT images, which provide anatomical information, but also use PET images, which provide the functional information needed to define the GTV. Physicians might take some other evidence or the historical record of the patient into consideration too. The procedure for physicians to define the GTV is complicated and professional. In principle, physicians cannot determine the GTV based on CT images only. In this study, our system undertakes GTV segmentation based only on the CT gray level. Unsurprisingly, the error in estimating the GTV was much larger than that of normal organs as a result.

Selecting the appropriate ROI can reduce the computation time in the image segmentation process. Our experiment, utilizing the same seeds with the RW algorithm, has the advantage of reproducibility. Another advantage of this system is that the user could utilize the GUI to increase or decrease the number of seeds to improve the image segmentation results interactively. This function is an add-on procedure, which makes the software flexible for the user to refine the result. The development of the software system is not intended to replace Eclipse, but to act as an addition to the process of developing an RTTP, speeding it up.

## 5. Conclusions

In this study, we developed a reliable software system which was able to segment five normal categories, including the lungs, airway, spinal cord, body, and heart, with an interactive function. In our experiments on 25 cases, we found that the contours of the five normal targets were similar to physician’s manual drawings, with average Dice coefficients of 0.83. The computation time of our system in processing a patient was about 2 to 4 min. The contours achieved by our system can be readily transferred to Eclipse for the follow-up process. The physicians could significantly reduce their operation time and human load in designing RTTPs for lung cancer patients in the CMUH. Our future goal is to combine CTs with PET scans in defining the GTV after image registration since PET scanning is an important step in delineating GTVs in clinics.

## Figures and Tables

**Table 1:** The HU table.

Hounsfield Units	Tissue
20 to 40	Muscle, vessel, soft tissue
0	Water
−30 to −70	Fat
−400 to −600	Lung
−1000	Air

**Table 2:** Dice coefficients of six targets in dataset I.

Case No.	Lungs	Airway	Heart	Spinal Cord	Body	GTV	Computation Time (s)
1	0.938	0.779	0.872	0.770	0.851	0.514	83
2	0.923	--- ^1^	0.850	0.692	0.795	0.403	87
3	0.962	0.948	0.849	0.853	0.852	--- ^2^	151
4	0.921	0.906	0.866	0.767	0.914	0.514	195
5	0.934	0.686	0.945	0.835	0.842	0.284	85
6	0.947	0.918	0.880	0.748	0.859	0.719	115
7	0.942	--- ^1^	--- ^1^	0.715	0.901	0.754	139
8	0.953	0.905	--- ^1^	0.760	0.774	0.889	119
9	0.870	0.823	--- ^1^	0.605	0.899	0.759	213
10	0.943	0.893	0.849	0.718	0.799	0.520	186
11	0.946	0.908	0.932	0.770	0.855	0.381	149
12	0.976	0.855	0.915	0.840	0.819	0.204	119
13	0.950	0.948	0.912	0.812	0.776	0.406	88
14	0.935	0.837	--- ^1^	0.587	0.898	0.598	231
15	0.926	0.782	0.823	0.596	0.873	0.891	152
Mean	0.94	0.86	0.88	0.74	0.85	0.57	141
Std.	0.02	0.07	0.04	0.09	0.04	0.21	48

^1^ No manual delineations. ^2^ Tumor segmentation fault.

**Table 4:** System comparison.

		Dice Coefficient	FPR	FNR
Lung	Eclipse	0.995	0.007	0.001
	Our system	0.938	0.080	0.007
Spinal cord	Eclipse	0.792	0.508	0.038
	Our system	0.770	0.551	0.097

**Table 3:** Dice coefficients of six targets in Dataset II.

Case No.	Lungs	Airway	Heart	Spinal Cord	Body	GTV
1	0.899	0.865	0.919	0.695	0.807	0.814
2	0.892	0.785	0.876	0.728	0.853	0.868
3	0.865	0.745	0.896	0.799	0.850	0.829
4	0.926	0.850	0.816	0.689	0.863	0.822
5	0.890	0.817	0.858	0.649	0.824	0.915
6	0.899	0.819	0.000	0.725	0.815	0.851
7	0.903	0.907	0.886	0.634	0.846	0.686
8	0.906	0.757	0.895	0.756	0.831	0.682
9	0.895	0.810	0.826	0.737	0.847	0.695
10	0.904	0.767	0.854	0.687	0.852	0.639
Mean	0.90	0.81	0.78	0.71	0.84	0.78
Std.	0.02	0.05	0.26	0.05	0.02	0.09
